# A non-randomized feasibility study of a voice assistant for parents to support their children’s mental health

**DOI:** 10.3389/fpsyg.2024.1390556

**Published:** 2024-07-31

**Authors:** Sally Richmond, Mietta Bell, Dyung Ngo, Marie B. H. Yap

**Affiliations:** ^1^Turner Institute for Brain and Mental Health, School of Psychological Sciences, Monash University, Melbourne, VIC, Australia; ^2^CogniVocal Pty. Ltd., Melbourne, VIC, Australia; ^3^Melbourne School of Population and Global Health, University of Melbourne, Melbourne, VIC, Australia

**Keywords:** voice assistant, mental health, artificial intelligence, parenting, feasibility

## Abstract

**Background:**

Mental disorders affect one in seven Australian children and although effective, evidenced based treatments exist, there is a critical shortage of mental health clinicians which has created a “treatment gap.” Artificial intelligence has the potential to address the high prevalence rates of mental disorders within overburdened mental health systems.

**Methods:**

This was a non-randomized feasibility study to evaluate the novel application of voice technology to an evidence-based parenting intervention designed to support children’s mental health. We deployed an Amazon Alexa app to parents recruited from the community (*N* = 55) and to parents with children receiving psychological treatment (*N* = 4). Parents from the community used the app independently whereas parents from the clinical group used the app in conjunction with attending a six-week parenting program. The primary outcome measure, feasibility was assessed in terms of acceptability, via recruitment and retention rates, quantitative surveys and qualitative interviews.

**Results:**

In the community group, the recruitment rate was 23.8% and the retention rate 49.1%. In the clinical group, all 6 families approached for recruitment agreed to participate and 4 out of 6 completed the trial. Parents attending the parenting program spent on average, three times longer using the app than parents from the community. Overall, parents reported that the app contained easy-to-understand information on parenting, and that they could see the potential of voice technology to learn and practice parenting skills. Parents also faced several challenges, including difficulties with installation and interactions with the app and expressed privacy concerns related to voice technology. Further, parents reported that the voices used within the app sounded monotone and robotic.

**Conclusion:**

We offer specific recommendations that could foster a better voice assistant user experience for parents to support their children’s mental health. The app is highly scalable and has the potential to addresses many of the barriers faced by parents who attempt to access traditional parenting interventions.

## 1 Introduction

Recent advances in spoken language technology, artificial intelligence, and conversational interface design, coupled with the emergence of smart devices, have increased the possibilities of using conversational interfaces for mental health (Abd-alrazaq et al., 2019; Meadows et al., 2020; Bérubé et al., 2021). The technology that responds to voice commands is referred to using many different terms, including voice assistant, virtual assistant, and conversational agent, but here the term “voice assistant” is used to encompass the above terminology and refer to voice-based interface for a smart speaker or phone. In the field of mental health, voice assistants are considered to have uses across many domains including the development of prediction (e.g., prediction of first episode psychosis in at-risk adolescents) (Bedi et al., 2015), detection/diagnosis and treatment (e.g., counseling) (Kim et al., 2021) and solutions for mental health care, however empirical evidence is scarce (D'Alfonso, 2020). This paper aimed to explore how voice assistants can be used by parents to support their children’s mental health.

Mental disorders affect one in seven Australian children and, if left untreated, can have a profound impact on a child’s development (Lawrence et al., 2015; Knapp and Wong, 2020). In developed countries the prevalence of child and adolescent mental disorders has remained relatively unchanged over the past 20 years despite (a) increased investment in mental health services (Jorm et al., 2017); and (b) the development of effective, evidence-based interventions (Lawrence et al., 2015). This is because many children with mental disorders do not access professional care (Hiscock et al., 2020; Mulraney et al., 2020). Parents play a critical role in supporting their children’s mental health but are often unable to access the required professional support due to extensive waiting times, costly treatment, and fears that their child will be stigmatized (Westrupp et al., 2020). Mental health systems are not equipped to support the diverse needs of people living with psychological distress or their families and carers (State of Victoria, 2021; Islam et al., 2022). Parents need affordable, timely access to evidence-based treatments to facilitate early intervention and mitigate the long-term consequences of childhood mental health problems.

Difficulties with emotional competence underpin many of the most common mental disorders in childhood (State of Victoria, 2021; Islam et al., 2022). Emotional competence includes the skills of emotion recognition, emotion expression, and emotion regulation (Mathews et al., 2016; Housman, 2017). Interventions aimed at improving emotional competence in children and subsequently reducing the prevalence of mental disorders, significantly improve well-being and educational outcomes (Goodsell et al., 2017). Families play a central role in child mental health and development (Havighurst et al., 2009; Schwartz et al., 2016; Phua et al., 2019; Wu and Lee, 2020). Given that many parental factors are modifiable, they can serve as key targets for mental health interventions. Parent emotion socialization describes the way parents respond to their children’s emotions and plays a critical role in the development of children’s emotional competence (Breaux et al., 2022). When parents coach their children’s emotions, children have increased emotional intelligence, improved social skills and academic results, and fewer behavior problems (Gottman, 1997; Johnson et al., 2017).

Currently parents can learn Emotion Coaching by attending a face-to-face parenting program, or through books or online courses. Tuning in to Kids is an example of a parenting program that focuses on developing supportive, emotionally responsive parenting through teaching the steps of Emotion Coaching (Havighurst et al., 2009). Tuning in to Kids has been evaluated in multiple randomized controlled trials and is demonstrated to decrease behavioral difficulties and reduce externalizing and internalizing difficulties for children and adolescents (Kehoe et al., 2014; Lauw et al., 2014; Havighurst et al., 2015). Despite this efficacy, parents often have anxieties about group participation, a sense of shame related to attending a parenting group or may not be able to afford the cost of attending all of which limit the reach and effectiveness of Emotion Coaching interventions (Finan et al., 2018; Westrupp et al., 2020).

Technology can overcome many of the barriers that prevent parents from engaging in parenting programs (Finan et al., 2020). Technology-based parenting interventions have shown positive effects on parenting and the emotional wellbeing of parents and children (Flujas-Contreras et al., 2019). Existing programs, such as Triple P (Sanders, 2019) and Parent–Child Interaction Therapy (Eyberg et al., 2001), have been modified to include online delivery (Flujas-Contreras et al., 2019). Attendance and participation are often higher for technology-based treatments (approximately 60–80%), compared to traditional parenting interventions (30–50%) (Flujas-Contreras et al., 2019; Hansen et al., 2019). Voice assistants represent a unique opportunity to transform parenting interventions by incorporating verbal interactions into existing digital offerings, including videos, quizzes and podcasts.

Voice assistants (e.g., Amazon Alexa, Google assistant) respond to voice commands using natural language programming to understand human language (Hoy, 2018). They can assist users with a range of tasks, such as playing music, checking weather forecasts, setting alarms and daily planning prompts (Cuadra et al., 2021). As speech is one of the most natural ways of human communication, using speech to interact with devices can lower the barriers of technology use for those with less familiarity or with fine motor and/or vision-related issues that make typing- and screen-based interfaces challenging. While voice assistants are not yet intended to replace human clinicians, through simple conversations they can interact with users to provide on-the-spot support (D'Alfonso, 2020). As a result, the role of artificial intelligence in the field of mental health is generating a significant amount of interest. A review focused on voice assistants for children, found they have been applied to support children’s learning, are able to be adapted for children with differing abilities, and can facilitate communication between a parent and child in the home environment (Garg et al., 2022). However, only three papers included in the review involved children with mental health or neurodevelopmental conditions.

This study is a novel application of voice technology to an evidence-based parenting intervention to support children’s mental health. We aimed to evaluate the feasibility of a voice assistant to improve parental knowledge of emotionally responsive parenting. Feasibility, as defined by the Bowen et al. framework (Bowen et al., 2009) was assessed in terms of acceptability, i.e., how the intended recipients reacted to the voice assistant for: 1. a community group of a parents who used the voice assistant independently; and 2. parents with children receiving psychological therapy who used the voice assistant in conjunction with attending a group parenting program.

## 2 Method

This non-randomized feasibility study evaluated the application of voice technology to an evidence-based parenting intervention. This study comprised two phases and was approved by the Monash University Human Research Ethics Committee (MUHREC ID 23249). A mixed-methods approach was used, including a survey and semi-structured group interviews. Study findings were reported based qualitative, COREQ (Tong et al., 2007), and intervention description, TIDieR (Hoffmann et al., 2014) guidelines (Supplementary Tables S1, S2).

### 2.1 Participants

Written consent was obtained from parents in each phase. In Phase 1 parents listened to the 30-min Phase 1 app on their existing technology (for example, a smart phone or smart speaker) over a two-week period in their own time. Phase 1 parents (*N* = 55) were recruited from the community via social media advertisements (e.g., Facebook and Instagram) and were (a) fluent in English; (b) had a primary school aged child/ren (*M*_child_ = 8.9 years, SD_child_ = 2.1 years); and (c) had access to a smart phone/speaker and the internet. Participants were mostly mothers (87.1%, *n* = 31) and located in the state of Victoria (90.1%, *n* = 43).

Phase 2 parents attended an online Tuning in to Kids parenting program (Havighurst et al., 2009, 2010) and each week listened to a parenting activity on the Phase 2 app. Phase 2 participants (*N* = 4) were invited to participate in the research via an email from administration staff at the Melbourne Children’s Psychology Clinic Hampton and were (a) fluent in English; (b) had a primary school aged child (*M*_*c*hild_ = 9.22 years, *SD*_child_ = 0.88 years); and (c) their children were receiving psychological treatment at the Melbourne Children’s Psychology Clinic Hampton. Based on parent reported symptoms, the children had a minimum of one elevated score for depression, anxiety or disruptive behaviors; the parents were experiencing mild levels of stress (Table 1). The Tuning in to Kids program was to be delivered in-person, however was moved online due to increasing COVID-19 restrictions in the city of Melbourne (Australia). When the online program began in 2020, Melbourne residents were required to comply with strict lockdown restrictions that required parents to work from home and home-school children (Auton and Sturman, 2022). Two participants withdrew from the parenting program prior to attending any sessions due to difficulties managing these demands.

**Table 1:** Phase 2 participant characteristics (*N* = 4).

	*M*	*SD*	*T*-score and/or qualitative descriptive range
Parent depression^a^	7.0	8.1	“Normal”
Parent anxiety^a^	5.0	6.2	“Normal”
Parent stress^a^	18.5	8.1	“Mild
Child depression (parent-reported)^b^	21.5	2.4	66; “Elevated, more concerns than typically reported”
Child anxiety (parent-reported)^c^	36.8	13.4	64; “Elevated”
Intensity of child disruptive behaviors (parent-reported)^d^	138.7	10.8	“Clinical range”
Number of child disruptive behaviors^d^	19	6.7	“Clinical range”

*T*-scores are standardized scores where the mean is equal to 50.^a^Depression Anxiety Stress Scales (DASS-21) subscale; ^b^Children’s Depression Inventory (CDI-2) total score; ^c^Spence Children’s Anxiety Scale (SCAS) total score; ^d^Eyberg Child behavior Inventory (ECBI) subscales.

The sample size was based on practical considerations, including participant flow, budgetary constraints and, as discussed, COVID-19 restrictions. Furthermore, considering this was an initial evaluation study to provide proof of concept and evidence for the intervention, and did not perform formal hypothesis testing for effectiveness or efficacy, a power analysis was not required (Eldridge et al., 2016).

### 2.2 Voice app

The Phase 1 and 2 variants of the app were developed by CogniVocal and built on the Amazon Alexa platform. The app utilized Amazon-proprietary Automatic Speech Recognition (ASR) and Natural Language Understanding (NLU) to interpret what the participants were saying, and customized Natural Language Processing (NLP) to provide meaningful and appropriate responses to the participants.

Details regarding the protection of participant data when interacting with the app was provided in the explanatory statement (Supplementary Figure S1). Participants were informed that interactions with the app were encrypted and stored on Amazon’s cloud. In general, the recordings were not accessible by either Monash University or CogniVocal. The responses to certain activities within the app, however were captured and this was stated by the app before the activity began. Participants were provided with instructions on how to review, listen and manually purge all recordings.

Participants accessed the Phase 1 and 2 variants of the app via invitation to a private beta testing module on the Alexa Skills Console. The first time parents accessed the app, they were asked to acknowledge verbally (“yes” or “no”) a disclaimer that the content was not a substitute for professional health and if they had any concerns to contact a health provider. To track use of the app (Phase 1 and 2), each participant was given a participant number and the app asked them to state this number verbally the first time they used the app. Parents could leave the app at any time by stating “Alexa, stop.” Parents could also navigate within the app to previous activities.

#### 2.2.1 Phase 1 app

Participants were emailed instructions to install the Amazon Alexa app (Supplementary Figure S2). This required participants to create an Amazon Alexa account and provide the research team with the email address associated with the account. Participants were then registered as beta testers and a link to test the Phase 1 app with a unique participant number was emailed to them. Participants accessed the Phase 1 app by opening Alexa, selecting ‘I accept’ to the terms and conditions when prompted, and clicking the ‘enable to use’ button. Participants were then able to click the ‘home’ icon and begin testing the app by stating, “Alexa, open emotion coaching study.”

There were five activities for parents to choose from: (1) Children’s behavior, an introduction to how Emotion Coaching can be used for challenging behaviors and the evidence-based benefits. (2) Emotion Coaching, an explanation of the five steps of Emotion Coaching: noticing your child’s emotions; seeing emotional moments as opportunities for connection and/or teaching with your child; listening and empathizing with your child; supporting your child to label their emotions; if required, problem solving. Two scenarios were available to highlight the differences between (3) dismissive parenting and (4) Emotion Coaching. (5) A five-question quiz to test knowledge of Emotion Coaching. To illustrate, the first question was “Can you tell me what the first step of emotion coaching is?” The user was given three response options and instructed to say “a,” “b” or “c.” In general, for each activity the level of voice interaction required from the parents was one-word responses, including “yes,” “no” and “repeat,” to prompts from the voice app at regular intervals. Parents could also request specific topics by stating the name of the activity, e.g., “Children’s behavior.” Each activity was approximately 5 min in duration, and the total phase 1 app time was no longer than 30 min if a parent listened from start to finish. Parents were given 2 weeks to listen to the Phase 1 app before they were sent the Phase 1 questionnaire.

#### 2.2.2 Phase 2 app

The instructions for accessing the Phase 2 app were broadly the same as detailed above for the Phase 1 app. Phase 2 participants could use their own technology to listen to the app and they were also provided with an Amazon Alexa Echo (smart speaker). The speaker was theirs to keep at the end of the program. Parents attended a six-week, two-hours per week, online Tuning in to Kids parenting program from October to November 2020. The program followed the content as prescribed in the Tuning in to Kids manual, however for the first 15 min of sessions two to six parents were interviewed about their experience with the app activity for that week and this discussion was audio recorded for qualitative analysis (see below). The interview guide (Supplementary Table S3) comprised open-ended question and explored the usability and acceptability of the app. The group interviews were conducted by the first author (SR), an accredited, female clinical psychologist with a Master of Clinical Psychology and PhD. Dr. Felicity McFarlane, Clinical Psychologist and Ms. Olivia Mort, Clinical Psychology Registrar, the Tuning in to Kids co-facilitators, and Ms. Mietta Bell, Research Assistant, were present for the interviews (no other team members were in attendance). The interview guide was reviewed (by MB) after the first interview to ensure relevant responses were drawn, additional prompts were not required. The group interviews were aimed at facilitating interactions between the interviewer and participants, i.e., not between participants, and focused primarily on investigating the acceptability of the Phase 2 App (Willemsen et al., 2023). The interviewer has previously conducted qualitative and quantitative research with parents and their children. Prior to study commencement, one of the Phase 2 participants had a professional relationship with the interviewer (SR). The participant’s child had been provided with psychological treatment by the interviewer in her capacity as a clinical psychologist. No relationship was established with the other Phase 2 participants prior to study commencement. None of the participants knew personal details about the interviewer. The professional characteristics of the interviewer, including her role as a clinician-researcher were detailed in the Parent/Guardian Information Statement and Consent Form which was signed by all participants. Handwritten notes were taken for each Tuning in to Kids sessions, including the interviews, and discussed amongst the co-facilitators in weekly clinical supervision sessions.

Activities were provided from Week 1 to 5 and were released every 7 days to coincide with the Tuning in to Kids group session. Except where noted, the content was based on the Phase 1 app. Week 1 contained information on parenting through COVID-19 with an emotion coaching perspective. Week 2 contained two activities, one about Emotion Coaching and the other about Children’s behaviors. Week 3 contained two activities to highlight the differences between a dismissive parenting approach and Emotion Coaching in the context of bedtime and a short quiz. Week 4 contained a guided interaction, where parents were encouraged to practice emotion coaching with Alexa. Alexa provided a scenario and asked parents to make a statement for each of the five steps of Emotion Coaching. For example, “Let us start the role play … say something to help your child recognize how they are feeling.” If the parent used one of the predefined “emotion words” Alexa would acknowledge the response and move to the next step, and if the response was not recognized Alexa would make a suggestion. Week 5, was another guided interaction based on a child’s frustration with home-schooling. Weeks 4 and 5 were designed to elicit the highest level of interactions with the difference being voice actors were used for the role of the mother and the child for the Week 5 scenario. The Week 4 and 5 activities were designed after the Phase 1 study was completed, hence this level of interaction was not a feature of the Phase 1 app.

### 2.3 Measures

#### 2.3.1 Phase 1 and 2: user experience survey

Parents answered a 17-item online questionnaire at the end of the evaluation period. The opening question stated, ‘If you were not able to test Emotion Coaching app it would be very helpful for us to know why. If you were not able to test please feel free to only complete this question.’

The following seven questions asked about parents’ prior experience with voice assistants, including the type and preference of product, e.g., Siri or Alexa, length of experience, location of voice assistant/s, likes and dislikes regarding voice assistants. The remaining questions were about the voice app: the type of device used to listen to the app; the clarity of the set-up instructions; the ability of the app to understand user voice commands; likes and dislikes regarding the app; suggested changes and general feedback.

#### 2.3.2 Phase 2 measures

##### 2.3.2.1 Depression anxiety stress scales

The Depression anxiety stress scales (DASS-21) (Lovibond and Lovibond, 1995) is a 21-item self-report questionnaire comprising three subscales of seven items each which assess depression, anxiety, and stress, respectively. Respondents are required to indicate to what extent each of the statements apply to them over the past week using a 4-point Likert scale ranging from ‘did not apply to me at all’ to ‘applied to me very much, or most of the time. Scores on each item are summed to produce a total score for each subscale, with higher scores indicating greater depression, anxiety, and stress, respectively, for each subscale (Lovibond and Lovibond, 1995). The DASS-21 also has good convergent validity (Page et al., 2007), with each subscale correlating strongly with other measures of depression, anxiety, and stress, respectively (Norton, 2007).

##### 2.3.2.2 Children’s depression inventory – parent report (CDI-2)

The CDI-2 (Kovacs, 2011) is a 17-item parent-report questionnaire which assesses the severity of depressive symptoms among children and adolescents. Parents are required to respond to items based on observations of their child over the past week using a 4-point Likert scale ranging from ‘Not at all’ to ‘Much or most of the time’. There are two subscales, Emotional Problems and Functional Problems, with scores on each item summed to produce a raw total score which can be standardized into *T*-scores. The CDI-2 is suitable for use with parents of children and adolescents aged 7–17 years and has good internal consistency and good discriminative validity (Bae, 2012; Kovacs, 2015).

##### 2.3.2.3 Spence Children’s anxiety scale – parent report (SCAS-P)

The SCAS-P (Spence, 1998) is a commonly used rating scale which assesses parent-report of child anxiety symptoms over the past week. It has 38 items rated on a 4-point Likert scale ranging from ‘Never’ to ‘Always’, broken into six subscales related to Social Phobia, Generalized Anxiety Disorder, Obsessive-Compulsive Disorder, Separation Anxiety Disorder, and Fear of Physical Injury, respectively. Each subscale is scored individually and summed to produce a total score which reflects overall symptoms of anxiety. The SCAS-P is suitable for use with parents of children aged 7–17 years and has good psychometrics (Arendt et al., 2014; Wang et al., 2016; Magiati et al., 2017).

##### 2.3.2.4 Eyberg child behavior inventory

The Eyberg child behavior inventory (ECBI) (Eyberg and Pincus, 1999) is a widely used 36-item parent-report measure of externalizing behavior problems in young children. Parents are required to respond to items using a 7-point Likert scale ranging from ‘1, Never’ to ‘7, Always’ and indicate whether the behavior is a problem using ‘Yes’ or ‘No’. Two scores are produced, an Intensity Score which assesses the frequency with which the behavior problems occur, and a Problem Score which assesses the total number of behaviors parents reported as problematic. The ECBI is suitable for use with parents of children aged 2–16 years and has established psychometrics (Abrahamse et al., 2015; Weeland et al., 2018).

### 2.4 Data analysis: acceptability

#### 2.4.1 Quantitative data

Descriptive statistics were applied to describe the characteristics of the sample by group (means (SD), percentages). Acceptability was evaluated in terms of recruitment rates, retention rates and the User Experience Survey data. Those lost to follow-up were not included in the analysis (*n* = 16).

#### 2.4.2 Qualitative data, phase 2

Acceptability was also evaluated based on the interview data using inductive thematic analysis using NVivo 20 (Braun and Clarke, 2006). Inductive thematic analysis was chosen as it emphasizes an exploratory approach to investigating conceptual themes (Braun and Clarke, 2006). Each interview script was analyzed with open coding by the first author (SR) to identify the significant themes or factors that emerged in the data based on the unit of analysis, acceptability. Next, the emerged themes were continuously discussed with a member of the research team (MB) until no new information was anticipated (Braun and Clarke, 2021). The example below demonstrates one participant’s lack of time to interact with the voice assistant. This response was coded as “No time.” Quotes are notated as follow, P# refers to the #th participant, and S# refers to the #th group session. For example, P5S2, is an excerpt from participant 5 in the second group session.

[No time] “it’s been a perfect storm because I’ve just found that the last 3 weeks have been chaotic … and so it probably has not made it into my top 3 which has been quite frustrating” [/No time] (P5S2).

We extracted all quotes from the five sessions to identify themes concerning use of the Emotion Coaching App. Initial themes were formed and we then applied thematic coding to all the qualitative data to strengthen and finalize the themes. This enabled identification of emerging themes in the data and allowed us to uncover perceptions and barriers that prevented or deterred access, understanding, and adoption, complementing our quantitative findings.

## 3 Results

### 3.1 Phase 1

#### 3.1.1 Recruitment and retention

**Figure 1:**
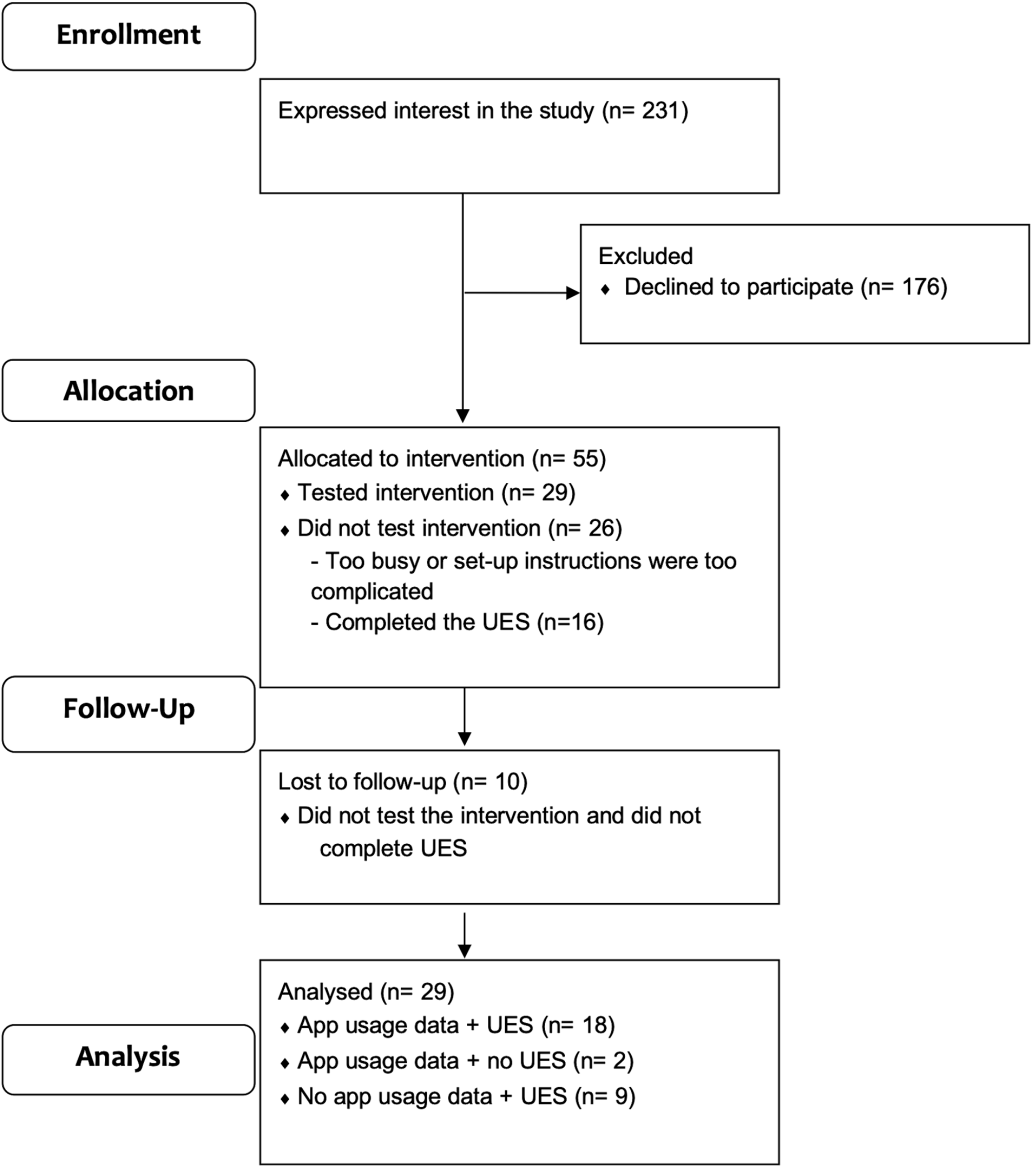
Phase 1 participant flow. UES = User Experience Survey.

Following social media advertising, 231 participants expressed interest in the study. All participants were emailed a link to the Phase 1 explanatory statement and consent form. In total, 176 participants declined to participate by either not responding to study emails (*n* = 167) or by requesting to be withdrawn (*n* = 9). A maximum of three contact attempts were made per participant and the mode of contact was dependent upon the information provided (i.e., email or phone). Of the 9 participants that withdrew, the majority quoted a lack of time as their reason. See Figure 1 for Phase 1 participant flow.

Fifty-five participants were eligible and consented to participate (23.8% recruitment rate), however 26 did not test the Phase 1 app. Of these participants, 16 provided feedback on their experience via the first question of the User Experience Survey. Most commonly, parents who did not use the Phase 1 app stated that the set-up instructions were too complex and difficult to follow or had other technical difficulties, such as creating an Alexa account (*n* = 11). Others indicated that they were too busy with family and/or home-schooling commitments (*n* = 5). Victorian children were being home-schooled during the study due to COVID-19 restrictions.

The Phase 1 app was tested by 29 participants, however two did not complete the User Experience Survey and therefore, 27 participants tested the intervention and completed the User Experience Survey (Figure 1). The retention rate was 49.1%.

Of the 29 participants who tested the app there were 3 subgroups: (1) 18 participants had app usage data stored against their participant number and they completed the User Experience Survey; (2) 2 participants had app usage data stored against their participant number and they did not complete the User Experience Survey; (3) 9 participants had app data that was not recorded (because their participant identification number was not recorded against their usage) and they completed the User Experience Survey.

Usage statistics, including the average time spent using the app, the average number of sessions and summaries for each activity are described in Table 2 for the 20 participants with app data.

**Table 2:** Phase 1 app usage (*N* = 20).

Total time Phase 1, all users (minutes)	438.87		
	*M* (*SD*)	Maximum	Minimum
Total time spent per user (minutes)	21.94 (15.73)	48.48	0^a^
Number of sessions	8.95 (7.70)	30	1
	Number of times accessed	Number of times completed (%)	
Children’s behavior	29	16 (55.17)	
Parenting scenarios	34	10 (29.41)	
Five steps of emotion coaching	45	20 (44.44)	
Quiz	29	5 (17.24)	

Includes 2 participants who did not complete the User Experience Survey.^a^User exited the app before they started an activity.

#### 3.1.2 User experience (quantitative)

As defined above, 27 participants tested the app and completed the User Experience Survey. Of those who used the Phase 1 app, 82% indicated that they already owned or used a voice assistant prior to the start of the study: 11 parents owned more than one voice assistant; seven owned Google Home only; four owned Siri only, and no participants indicated use of only Alexa. Parents indicated the thing they liked most about voice assistants was that they are quick, time saving and easy to use, their hands-free use was also a major benefit. Dislikes for voice assistants included the inability of the voice assistants to understand voice commands and the need for repetition and others were concerned that voice assistants are always listening, and personal conversations could be recorded.

Half of the participants (*n* = 14, 52%) reported difficulties with the instructions for the Phase 1 app, ranging from descriptions of “confusing” and “overwhelming” to issues with stating the participant numbers. Suggestions for improving the instructions included making them more basic/step-by-step, having trouble-shooting information, having Android and Apple specific instructions, and including information about basic Alexa commands.

Most parents used their phones to listen to the app (*n* = 22; 80%), while others used smart speakers or tablets. When participants were asked if the Phase 1 app understood their voice commands, 70% reported yes or mostly yes (*n* = 19). Useful, helpful, and easy to understand information was endorsed as the most liked feature of the app (*n* = 19). The most common dislike was either the voice (monotone; too slow) and/or difficulties related to navigating the app.

### 3.2 Phase 2

#### 3.2.1 Recruitment and retention

Of the 6 participants that were invited to participate, all consented, and 2 participants withdrew prior to the start of the parenting group due to COVID-19 restrictions.

#### 3.2.2 User experience (quantitative)

Four participants tested the Phase 2 app. Usage statistics, including the average time spent using the app, the average number of sessions and summaries for each activity are described in Table 3.

**Table 3:** Phase 2 app usage (*N* = 4).

Total time Phase 2, all users (minutes)	303.63		
	*M* (*SD*)	Maximum	Minimum
Total time spent per user (minutes)	75.91 (58.31)	142.89	0.73
Number of sessions	26.25 (20.29)	50	1
	Number of times accessed	Number of times completed (%)	
Introduction	4	4 (100)	
Parenting COVID-19	5	4 (80)	
Children’s behavior	4	4 (100)	
Dismissive scenario (Bedtime)	4	4 (100)	
Five steps of emotion coaching	4	4 (100)	
Guided interaction	8	4 (50)	
Home-school guided interaction	20	8 (40)	
Quiz	6	4 (66.67)	

Of the four participants who tested the Phase 2 app, three User Experience Surveys were completed. Two participants already owned or used a voice assistant prior to the start of the study. Flexibility of using on demand and hands-free access were noted as likes for voice assistants. Dislikes were listed as privacy (and listening/data collection) and lack of understanding (voice recognition).

Two participants used the speaker provided to listen to the app and the others used a phone. Two of the three participants found the instructions difficult to follow. The fourth participant (who did not complete the User Experience Survey) also had difficulty with the instructions and received telephone installation support from CogniVocal. Suggestions for improving the instructions included making them more basic/step-by-step. When participants were asked if the Phase 2 app understood their voice commands, two reported yes or mostly yes. Likes included the innovation, the potential to practice role-plays in real time which was described as less confronting than practicing with a group, and the more interactive modules toward the end of the program. Dislikes included talking to a computer/synthetic voice, that the app is based on words but that there is no recognition of tone or pace of voice, and that there is no ability to incorporate non-verbals such as facial expressions.

#### 3.2.3 User experience (qualitative)

Three broad themes were identified in the data: benefits, challenges encountered when using the voice app; and criticisms of the content.

##### 3.2.3.1 Benefits

The benefits of the voice app were grouped into two sub-themes: the potential of the voice app as a new way to learn about parenting, and the usefulness of content itself.

“It has the potential to be able to practice, which I thought was really great” (P5S5).

“I’ve been thinking about whether it could be useful for kids to be able to activate…” (P5S4).

“with the role plays … there was some usefulness to it, it … made me pause and think” (P7S6).

The participants reflected on the different types of activities within the voice app.

“does a role play for you, which was really cool” (P5S4).

“the content is actually really good and the exercises are really good. The little polls and tests I found useful and helpful” (P7S5).

##### 3.2.3.2 Challenges

Within the theme of Challenges, four sub-themes emerged: difficulties interacting with a voice assistant (Alexa); using the wake word; being understood within the app; and overcoming privacy concerns. Parents encountered several challenges when they first started using the app. Initially, parents had difficulty installing the app onto their speaker or phone. Some of these initial difficulties were related to differing experience levels with voice technology and not directly related to the app itself.

“After jumping through hoops to get that (the voice app) set-up and everything, I tried but I got an error.” (P7S4).

“I could not even get Alexa to tell the weather, I could not even do that.” (P5S5).

“…what the commands are, what the trigger word are … that would make it a bit easier to use” (P5S5).

More specific to the app, some parents had difficulty with the wake word. To start the app, parents received instructions to say “Alexa, open emotion coaching assistant” or “Alexa, launch emotion coaching assistant.”

“I had trouble opening the app because I kept forgetting what to call it.” (P5S4).

“a mouthful every time, even just trying [to open it].” (P3S4).

In addition to these challenges, participants had difficulties with the number of steps required to set up and access the app. As described above, because the app was released in a testing state, participants had to register an email address and follow a link to gain access. As part of this process and to ensure participants could be identified, participants were given a unique number that they were required to state verbally the first time they used the app. Some participants had difficulty with these initial steps to test the app.

“I was using it and now I’m trying to get back out… to reset it under my ID number… I think I’ve just wasted a lot of time” (P2S5).

Participants reported that once they were within the app, that their voice commands were often not understood, and they inadvertently exited the app when that was not their intention.

“... would go to speak and was sort of interrupted by it [Alexa] repeating the piece again, and I think that happened a second time, and then we quit out.” (P3S6).

“It’s not listening.” (P3S2).

Privacy concerns and thoughts around being recorded were raised across three of the five sessions. Including feeling anxious at the thought of being recorded and someone listening to the content.

“I suppose we are also pretty security conscious, so we do not have Alexa.” (P5S3).

“I still have these … in the back of my mind because it’s going to record what you are doing and the and the privacy around that.” (P5S5).

“[participant] froze and I shut down completely … we tried to recover as best we could.” (P3S6).

##### 3.2.3.3 Criticisms of the content

Within the theme of Criticism of the Content three sub-themes emerged: amusing, monotone/unresponsive voice, difficult to navigate, and not interactive. The first two sub-themes were both related to the voice used in the app. In Weeks 1–2 the voice used was mostly the standard ‘Alexa’ voice. In Weeks 3–4 other Alexa (synthetic) voices were used to simulate the scenarios between children and parents. For Week 5, amateur voice actors were used for the parent and the child.

“Alexa’s attempt at different voices is very amusing” (P3S4).

“… a computer kid and so we were just in fits of laughter” (P5S4).

“Sounds a little bit mechanistic…they are a robotic voice” (P7S5).

“It’s still a computer obviously … felt a bit stilted” (P5S2).

In general, the amateur voices that were used in Week 5 were well received by the participants.

“having that more natural voice … I think it worked a lot better” (P5S6).

“it was a warmer experience because it was a more natural and more human voice” (P7S6).

Parents experienced difficulty using verbal commands to move between exercises.

“It did take me a while to work out how to go back to week one because there’s nothing that tell you the command” (P7S5).

“I cannot find … go back to the beginning, so I’m sort of missed whatever she said at the beginning and then we are trying to do the exercise …having missed the first instruction” (P5S5).

The Phase 2 app was designed to become more interactive as participants moved through the content. Week 1 required the smallest number of verbal responses, i.e., the majority of responses were yes/no, okay. Different types of interactions were added across the weeks, e.g., when participants completed a quiz on emotion coaching, they were required to select the correct answer by stating “a,” “b” or “c.” As described above, Week 5 had the highest level of interaction between the participants and the app.

“Not interactive so at the moment I would probably choose a podcast over the app” (P3S3).

“I still … describe it like I’m on the ghost train as opposed to a normal conversation or role play that we are doing here” [in the group sessions] (P3S6).

## 4 Discussion

This study aimed to evaluate the acceptability of a voice assistant to improve knowledge of emotionally responsive parenting. The main findings are that: (1) parents were able to identify several benefits of using the app, including the helpful and easy to understand information on parenting, the interactive/conversational nature of the app and hands-free use. Phase 2 parents recognized the potential of the app to enable them to practice a parenting approach; (2) parents also faced several challenges including installation issues, using voice commands to interact with the app (e.g., Alexa commands), and privacy concerns related to voice technology; (3) parents in both phases experienced the voices as computer generated and robotic.

Overall, participants found the parenting information provided by the app useful, helpful and easy to understand. In terms of the content of the app, Emotion Coaching is a parenting approach typically delivered in group programs (e.g., Tuning in to Kids) or through training programs (e.g., Gottman Institute, videos and online). In those contexts, there is a body of research to support it as an effective parenting intervention for improving children’s mental health and/or behaviors (Kehoe et al., 2014; Havighurst et al., 2015; Goodsell et al., 2017; Johnson et al., 2017). The purpose of the study was to investigate the delivery of the information in a different way. Therefore, that the majority of Phase 2 participants found the information contained in the app to be useful and easy to understand, suggests that the app maintained fidelity with the content of evidence-based Emotion Coaching programs, however a direct comparison is required to evaluate fidelity.

The interactive/conversational aspect of the app was also identified as a positive feature by both groups of participants, as was the hands-free use. Similar benefits have been reported by other users of voice assistants. Hands-free use, for example, has also been reported in groups of people with neuromuscular problems (Alonso et al., 2021). The conversational nature of voice assistants is inherently associated with human-like properties, which can lead users to personify the device. This can have a positive or negative effect on user experience. In a study of older adults, with a simple question and answer format, approximately 12% of the voice commands had non-functional phrases, for example, the users asked the voice assistant about its human-like characteristics (Kim and Choudhury, 2021). In this study, recordings were only taken for one of the activities (home-schooling guided interaction) and therefore the level of personification behaviors was not assessed. Overall, the results demonstrate that parents were able to identify several benefits of the voice app.

One of the major differences between the Phase 1 and Phase 2 apps was the increased level of interaction for the parents who were using the app in conjunction with attending a Tuning in to Kids program. A major component of the Tuning in to Kids program is using role-plays to practice the steps of Emotion Coaching. The Phase 2 app was further developed from the Phase 1 version to include this type of learning with a voice assistant. Several of the parents recognized the potential to practice emotion coaching using the app, at a time that was convenient for them. It is also likely that practicing with a voice assistant could be less confronting than practicing with a group, another potential benefit. This may explain the increased use by Phase 2 parents (approximately 3.5 times more) compared to Phase 1, however Phase 2 parents used the app over a longer 6-week period, whereas Phase 1 parents evaluated over a 2-week timeframe. Phase 2 parents also had a relevant context for the using the app (i.e., attendance at the Tuning in to Kids program), they were held accountable for their usage at the start of each group session, and their children were experiencing psychological distress and/or behavioral challenges. Hence, increased use by the Phase 2 parents may have also been related to greater motivation to support their children.

One of the major challenges experienced by over 50% of participants was difficulty with the set-up instructions for the app. The decision to keep the app in testing mode for the duration of the study was a contributing factor. This decision provided flexibility if any major bugs were discovered, however it added steps and complexity for users. Users could not simply download the voice app from the Alexa store but had to be enabled as beta testers via a number of emails. The complexity of the set-up instructions was a significant barrier for those who did not test the Phase 1 app. For the Phase 2 users, challenges with the set-up were less pronounced because the participants had an opportunity in the group sessions to problem solve. In addition, to link the user’s activity on the app to the study, participants were provided with a number. The voice app asked for the number to be verbally stated the first time the user opened the app. Several users had difficulty having their number recognized and were able to by-pass this step, subsequently the usage data for nine Phase 1 participants was not recorded correctly. More broadly, research indicates user authentication for voice-based services needs to be a compromise between security and ease of use, with the spoken PIN method one of most common solutions (Renz et al., 2023). It is recommended that future studies ensure there is a simple and reliable way to identify participants and conduct thorough pre-study testing to avoid missing data.

Another challenge experienced by some users (Phase 1 and 2) was that they were unfamiliar with how voice assistants work. Many parents did not even have a basic understanding of how to interact with a voice assistant. This was also evident in difficulties using the wake word to activate the app (“Alexa, open …). Within the app, this limited understanding manifested as difficulties in navigation (e.g., moving to different activities) and created repetition for the users where they reported that they were listening to the same information because they could not find the correct words to move on or interrupt Alexa. Users were not aware that they could say “Alexa, stop” at any time or “Go to …” to move to a certain activity. It is likely that more time orientating users to the voice assistant prior to using the app may have improved their experience. It is recommended that users are provided with simple and clear: basic commands for interacting with a voice assistant prior to voice app use; and written and visual (e.g., video) instructions for accessing a voice app for all relevant devices (e.g., smartphone Android and iOS, smart speaker, etc.). Technical difficulties may also explain the reduced use of smart speakers in the study. In Phase 1 most parents used their phones to listen to the app and in Phase 2, although all participants were provided with a smart speaker, two participants used their phone. Furthering understanding of user preference for a smart speaker or smart phone to interact with the app would enable instructions to be more tailored to improve the likelihood of successful installation.

Parents also shared privacy concerns about their conversations being recorded and shared without their knowledge. When operational, voice assistants need to be in a continuous listening-mode to recognize the designated wake-word. The ‘always-listening’ feature of voice assistants is responsible for the hands-free convenience of the technology but also has privacy implications. There is the potential for the device to reveal private information about the user or to misinterpret a non-wake word and record without the user’s knowledge (Pridmore et al., 2019; Barceló-Armada et al., 2022). It is recommended that users are provided with accessible information on privacy and voice assistants to overcome barriers to engaging with this type of technology. Privacy in the context of voice assistants is complex however and different users have different privacy concerns. For example, in the UK, user concerns about third parties listening to recordings are common however privacy protection behaviors, including turning off the voice assistant and reviewing and deleting the information collected, are not (Lutz and Newlands, 2021). More specific to the field of mental health, recent reviews have concluded that if AI is to be used to augment care, steps must be taken to ensure privacy with regulation compliant and acceptable protections, e.g., patient data must not be shared with third parties without proper consent (King et al., 2023; Pandya et al., 2023). It seems likely that AI models for health care may be required as opposed to generalized AI software (King et al., 2023). In addition, ethical and legal standards need to be developed for AI use, how to disclose and inform about this use and its validation in clinical environments (King et al., 2023; Pandya et al., 2023). Taken together, protecting patient privacy remains a key concern in the use of AI in health care.

As discussed above, parents saw the conversational aspect of the app as a benefit however they consistently experienced the voice used within the app as monotone and robotic. Although, in general the robotic sound of the voices used in the app were discussed as a negative by participants, this point is still under debate within the literature. Some researchers suggest that for people without extensive understanding of technology, a robotic voice may remind them that they are not speaking to a human and help them recognize the limits and not overestimate the voice assistant’s capabilities (Schreuter et al., 2021). Others state that making AI sound more human-like strengthens the human-AI interaction by increasing trust. Emerging research indicates that there is no difference in the level of conformity (operationalized as getting users to change their minds on a quiz) between a human voice and a robot voice (Schreuter et al., 2021). This is an important consideration, particularly in the field of mental health as too much trust in a voice assistant could be problematic if, as an extreme example, a person with severe mental ill health overestimates the ability of digital intervention and does not seek treatment from a face-to-face service when this is warranted. Parents also described the Alexa voices used in the app as monotone. Humans can find the Alexa voice to be emotionally expressive, however this effect has been observed when emotionally expressive Alexa interjections are used (e.g., “Awesome!”) compared to the neutral production generated by the default Alexa Voice (Cohn et al., 2021). It is recommended that future research explores the use of emotionally expressive and responsive voices in the application of voice apps to the field of mental health.

The findings of this study demonstrate a mix of benefits and challenges/concerns that vary across the participants. This complexity reflects a recent study of American adults’ attitudes toward AI, that found different segments within the population including the negative, perceiving risks outweighing benefits; the ambivalent, seeing high risks and benefits; the tepid, perceiving slightly more benefits than risks; the ambiguous, perceiving moderate risks and benefits; and the indifferent, perceiving low risks and benefits (Bao et al., 2022).

This study is a novel exploration of voice technology use by parents to support their children’s mental health. Google has suggested that parents use voice assistants more than non-parents as part of their daily routine and for multitasking (Kleinberg, 2018). Further, parents play a key role in supporting their children’s mental health (Yap and Jorm, 2015). Taken together, families are an important environment where voice assistant technology may be useful. A strength of this study is that parents interacted with the voice app and associated device in their own usual environment. If we had conducted this study in a controlled lab setting the results would not have accurately represented the interactions within the participants’ lives. The study findings for Phase 2 were limited by the small number of group members. Recruitment and attendance at the Tuning in to Kids program was impacted by COVID-19 restrictions and impacted by the decision to run the group online rather than face-to-face as originally planned. It is also possible that for the Phase 1 parents, receptiveness to digital mental health intervention may have shifted during the COVID-19 pandemic (Linardon et al., 2021).

In this study, the majority of participants who tested the app and completed the User Experience Survey already owned or used a voice assistant. This indicates prior experience with voice assistants may have been associated with testing the voice app. In technology-based interventions it is important to consider whether access to technology creates an enrolment bias (Toscos et al., 2019). As an example, participants in the study were excluded if they did not have access to a smartphone/speaker and the internet. Note that although Phase 2 participants were provided with a smart speaker, all participants needed access to the internet. Excluded participants may benefit from the app, potentially even more, and therefore identifying and addressing inequities will be important for future research. This also applies to consideration of users with impairments, for example voice technology could be helpful for parents with literacy difficulties. It will be important for future research to include a diverse range of users through co-design strategies, throughout the development process to ensure digital health solutions are usable, accessible and equitable (Henni et al., 2022). Co-design has also been identified as important for enhancing credibility, acceptance and uptake of digital interventions (Torous et al., 2018; Connolly et al., 2021).

## 5 Conclusion

In this study, parents could see the benefits and potential of interacting with voice technology to support their children’s mental health. There were, however, a number of challenges and therefore further development of the voice app is required to integrate the findings of the current study and assess feasibility.

## Data availability statement

The raw data supporting the conclusions of this article will be made available by the authors, without undue reservation.

## Ethics statement

The studies involving humans were approved by Monash University Human Research Ethics Committee. The studies were conducted in accordance with the local legislation and institutional requirements. The participants provided their written informed consent to participate in this study. Written informed consent was obtained from the individual(s) for the publication of any potentially identifiable images or data included in this article.

## Author contributions

SR: Conceptualization, Formal analysis, Funding acquisition, Investigation, Methodology, Supervision, Writing – original draft, Writing – review & editing. MB: Data curation, Investigation, Methodology, Writing – original draft, Writing – review & editing, Project administration. DN: Software, Writing – original draft, Writing – review & editing, Conceptualization. MY: Methodology, Supervision, Writing – original draft, Writing – review & editing.
