# Supplementary material for: A non-randomized feasibility study of a voice assistant for parents to support their children’s mental health
Source: Front Psychol. 2024 Jul 31;15:1390556. doi: 10.3389/fpsyg.2024.1390556 (PMC11323748; doi:10.3389/fpsyg.2024.1390556)

Supplementary Material

# Supplementary Figures and Tables

**Table S1:** COREQ Guideline - a 32-item checklist for qualitative studies

|  | No. Item | Guide Questions/Description | Page # |
| --- | --- | --- | --- |
| Domain 1: Research Team and Reﬂexivity | | | |
| Personal Characteristics | | | |
| Interviewer/facilitator | 1 | Which author/s conducted the interview or focus group? | 5 |
| Credentials | 2 | What were the researcher’s credentials? e.g., PhD, MD | 5 |
| Occupation | 3 | What was their occupation at the time of the study? | 5 |
| Gender | 4 | Was the researcher male or female? | 5 |
| Experience and Training | 5 | What experience or training did the researcher have? | 5 |
| Relationship With Participants | | | |
| Relationship Established | 6 | Was a relationship established prior to study commencement? | 6 |
| Participant Knowledge of The Interviewer | 7 | What did the participants know about the researcher? e.g., personal goals, reasons for doing the research | 6 |
| Interviewer Characteristics | 8 | What characteristics were reported about the interviewer/facilitator? e.g., Bias, assumptions, reasons, and interests in the research topic | 6 |
| Domain 2: Study Design | | | |
| Theoretical Framework | | | |
| Methodological Orientation and Theory | 9 | What methodological orientation was stated to underpin the study? e.g., grounded theory, discourse analysis, ethnography, phenomenology, content analysis | 8 |
| Participant Selection | | | |
| Sampling | 10 | How were participants selected? e.g., purposive, convenience, consecutive, snowball | 3-4 |
| Method of Approach | 11 | How were participants approached? e.g., face-to-face, telephone, mail, email | 3-4 |
| Sample Size | 12 | How many participants were in the study? | 8-9 |
| Non-Participation | 13 | How many people refused to participate or dropped out? Reasons? | 8 -9 Figure 1 |
| Setting | | | |
| Setting of Data Collection | 14 | Where was the data collected? e.g., home, clinic, workplace | 7 |
| Presence of Non-Participants | 15 | Was anyone else present besides the participants and researchers? | 5-6 |
| Description of Sample | 16 | What are the important characteristics of the sample? e.g., demographic data, date | 3-4 & Table 1 |
| Data Collection | | | |
| Interview Guide | 17 | Were questions, prompts, guides provided by the authors? Was it pilot tested? | Table S3 & page 5 |
| Repeat Interviews | 18 | Were repeat interviews carried out? If yes, how many? | Yes, 5 |
| Audio/Visual Recording | 19 | Did the research use audio or visual recording to collect the data? | 4 |
| Field Notes | 20 | Were ﬁeld notes made during and/or after the interview or focus group? | 5-6 |
| Duration | 21 | What was the duration of the interviews or focus group? | 5 |
| Data Saturation | 22 | Was data saturation discussed? | 8 |
| Transcripts Returned | 23 | Were transcripts returned to participants for comment and/or correction? | No |
| Domain 3: Analysis And ﬁndings | | | |
| Data Analysis | | | |
| Number of Data Coders | 24 | How many data coders coded the data? | 8 |
| Description of the Coding Tree | 25 | Did authors provide a description of the coding tree? | NA |
| Derivation of Themes | 26 | Were themes identiﬁed in advance or derived from the data? | 8 |
| Software | 27 | What software, if applicable, was used to manage the data? | 8 |
| Participant Checking | 28 | Did participants provide feedback on the ﬁndings? | No |
| Reporting | | | |
| Quotations Presented | 29 | Were participant quotations presented to illustrate the themes/ﬁndings? Was each quotation identiﬁed? e.g., participant number | 10-12 |
| Data and ﬁndings Consistent | 30 | Was there consistency between the data presented and the ﬁndings? | 8-12 & 12-15 |
| Clarity of Major Themes | 31 | Were major themes clearly presented in the ﬁndings? | 10 |
| Clarity of Minor Themes | 32 | Is there a description of diverse cases or discussion of minor themes? | 10-12 (sub-themes) |

* Loction on original submission (page number will be different on published version)

Tong A, Sainsbury P, Craig J. Consolidated criteria for reporting qualitative research (COREQ): a 32-item checklist for interviews and focus groups. Int J Qual Health Care. 2007;19(6):349-357.

**Table S2:** TIDieR Guideline – checklist of information to include when describing an intervention

| **Item number** | **Item number** | **Located, Page *** |
| --- | --- | --- |
|  | **BRIEF NAME** |  |
| **1.** | Provide the name or a phrase that describes the intervention. | 3 |
|  | **WHY** |  |
| **2.** | Describe any rationale, theory, or goal of the elements essential to the intervention. | 4-6 |
|  | **WHAT** |  |
| **3.** | Materials: Describe any physical or informational materials used in the intervention, including those provided to participants or used in intervention delivery or in training of intervention providers. Provide information on where the materials can be accessed (e.g. online appendix, URL). | 4-6 & Figure S1 |
| **4.** | Procedures: Describe each of the procedures, activities, and/or processes used in the intervention, including any enabling or support activities. | 3-6 |
|  | **WHO PROVIDED** |  |
| **5.** | For each category of intervention provider (e.g. psychologist, nursing assistant), describe their expertise, background and any specific training given. | 5-6 |
|  | **HOW** |  |
| **6.** | Describe the modes of delivery (e.g. face-to-face or by some other mechanism, such as internet or telephone) of the intervention and whether it was provided individually or in a group. | 3-6 |
|  | **WHERE** |  |
| **7.** | Describe the type(s) of location(s) where the intervention occurred, including any necessary infrastructure or relevant features. | 3-4 |
|  | **WHEN and HOW MUCH** |  |
| **8.** | Describe the number of times the intervention was delivered and over what period of time including the number of sessions, their schedule, and their duration, intensity or dose. | 4-6 |
|  | **TAILORING** |  |
| **9.** | If the intervention was planned to be personalised, titrated or adapted, then describe what, why, when, and how. | NA |
|  | **MODIFICATIONS** |  |
| **10.^ǂ^** | If the intervention was modified during the course of the study, describe the changes (what, why, when, and how). | NA |
|  | **HOW WELL** |  |
| **11.** | Planned: If intervention adherence or fidelity was assessed, describe how and by whom, and if any strategies were used to maintain or improve fidelity, describe them. | NA |
| **12.^ǂ^** | Actual: If intervention adherence or fidelity was assessed, describe the extent to which the intervention was delivered as planned. | NA |

* Loction on original submission (page number will be different on published version)

**Table S3:** Semi-structured interview guide

| **Focus point** | **Key questions and prompts** |
| --- | --- |
| 1. Technical issues and challenges | - Were you able to access the activities for this week? - Please describe your experience of accessing the app and the weekly activities. - What have the challenges been? - Is further support required? |
| 1. Content questions | - What did you think of the content for this week? - Did you enjoy/like/find valuable any of the content for this week? - Did you have any concerns/dislike/not enjoy any of the content for this week? - Are there any topics or information you would have liked to have seen in the app that we missed? Or any suggestions for improvements? |
| 1. Maintaining behaviours and strategies | - How confident are you to access the content for the following week? - Do you foresee any challenges moving forward? |
| 1. Closing | - Is there anything that you think would be important to mention that we haven’t covered? |

**Figure S1**: **Explanatory Statement**

| HREC Project Number: | 23249 | | |  |
| --- | --- | --- | --- | --- |
| Short Name of Project:  Full Name of Project: | A Voice Assistant for Parenting: Phase 1  “Hey Alexa”: Exploring the use of artificial intelligent voice assistants in a parenting program. | | |  |
| **Principal Researcher:** | Dr Sally Richmond, Monash University | | | |
| **Version Number:** | 1 | **Version Date:** | 4 May 2020 | |

Thank you for taking the time to read this Parent/Guardian Information Statement and Consent Form. We would like to invite you to take part in a research project that is explained in this form.

This form is 6 pages long. Please make sure you have all the pages.

What is an Information Statement and Consent Form?

An Information Statement and Consent Form tells you about the research project. It explains exactly what the research project will involve. This information is to help you decide whether or not you would like to take part in the research. Please read it carefully.

Before you decide if you want to take part or not, you can ask us any questions you have about the project. You may want to talk about the project with your family, friends or health care worker.

Taking part in the research project is up to you

It is your choice whether or not you take part in the research project. You do not have to agree if you do not want to. If you decide you do not want to take part, it will not affect the treatment and care your child gets at The Melbourne Children’s Psychology Clinic - Hampton.

Signing the form

If you want to take part in the research, please sign the consent form at the end of this document. By signing the form, you are telling us that you:

- understand what you have read
- had a chance to ask questions and received satisfactory answers
- consent to taking part in the project.

We will give you a copy of this form to keep.

It is estimated that around one in seven Australian children experience mental health issues and about half of all serious mental health issues in adulthood begin before the age of 14.

Parents can have a significant influence on their children’s emotional and behavioural development and their mental health. Tuning in to Kids is a parenting program that aims to support parents in teaching their children how to understand and regulate their emotions and as a result develop emotional competence. Tuning in to Kids has been shown to decrease challenging behaviours in young children and decrease internalising symptoms in adolescents. Although the program has had positive outcomes for families, the program can be challenging for some parents. Parents can have anxieties about group participation or may find activities that include expressing vulnerable emotions difficult. Technology may help overcome some of these issues.

Monash University, in partnership with CogniVocal (a company that designs voice interactions, cognivocal.com), is developing a voice assistant to support parents to learn about a parenting approach called, *Emotion Coaching*. The aim of the current project is to explore how parents use the voice assistant and to use this feedback to refine the voice assistant for future users.

1. **Who is running the project?**

The study is a joint study between Monash University, the Melbourne Children’s Psychology Clinic – Hampton, and CogniVocal . The research team for this study includes researchers and psychologists from Monash University and the Melbourne Children’s Psychology Clinic, and designers from CogniVocal. The protocol for the study was written by the research team at Monash University. This study is being funded by a Monash University Community Engagement Grant. The Melbourne Children’s Psychology Clinic – Hampton and CogniVocal, have donated some services and goods.

Dr Sally Richmond and Associate Professor Marie Yap are from the Turner Institute of Brain and Mental Health at Monash University. Dr Richmond is a psychologist at the Melbourne Children’s Psychology Clinic – Hampton and a research fellow. Dr Richmond has completed training in a parenting program based on Emotion Coaching (*Tuning in to Kids)*. Associate Professor Yap is a psychologist and international expert on research into digital parenting programs.

Dr Felicity McFarlane is a clinical psychologist and director of the Melbourne Children’s Psychology Clinic – Hampton. Drs Richmond, McFarlane, and Yap are registered with the Australian Health Practitioner Regulation Agency (AHPRA).

Mr Dyung Ngo is the chief technical officer at CogniVocal and Mr Andre Alcantara is the designer at CogniVocal.

The background intellectual property (IP) associated with the voice assistant is owned by Cognivocal. None of the research team have a financial interest in the voice assistant.

1. **Why is my child being asked to take part?**

We are inviting you participate in this project because you have a child aged between 6 and 11 years.

1. **What do I need to do in this project?**

The project is being run in two phases. The information contained in the document relates to the first phase. In Phase 1, you will be asked listen to the voice application on your existing technology (for example, your phone or smart speaker). After a 2-week period you will be asked to complete a short survey (online or hard copy) regarding the voice application. The survey should take no more than 15 minutes. You will also be invited to attend an optional focus group, with a maximum of 8 participants, at a convenient time. Focus group will be held at the Melbourne Children Psychology Clinic - Hampton or via video-conferencing software (if required to comply with COVID 19 restrictions) and will take approximately 30 minutes to complete. All focus groups and surveys will be completed over a 3-month period.

1. **Can I stop taking part in the project?**

You can stop taking part in the project at any time. You just need to tell us. You do not need to tell us the reason why. If you leave the project, we will use any information already collected unless you tell us not to.

1. **What are the possible benefits for my child and other people in the future?**

This project will help us assess whether voice assistants are feasible for use in parenting programs. It will also give families the opportunity to use and be involved in the design of a voice assistant. Whilst researchers hope to see improvements in the parents who use the voice assistant, it is possible that these improvements will not be seen and there will be no benefit.

1. **What are the possible risks, side-effects, discomforts and/or inconveniences?**

We do not anticipate any risks or side-effects from participation in this project. Parents may find the focus group session to be tiring but we will provide a short break half-way through each session. Parents will also be encouraged to take breaks when needed.

We do not expect the survey to cause any distress, however you can skip any questions that you do not want to answer.

Although we do not anticipate any risk associated with interacting with the voice assistant there are additional steps participants can take to minimise exposure to risk. These steps are outlined in sections 9 -13 of this statement.

If researchers notice a decline in your wellbeing during the focus group, they may provide you with a referral to an appropriate service for support and/or consult with the Principal Investigators to determine whether it is in your best interests to be withdrawn from the study. All participants will also be provided with a list of resources they can access for support. Parents can withdraw from the study at any time.

1. **What will be done to make sure my information is confidential?**

We respect your privacy; any information obtained in connection with this project will remain confidential. Only the researchers involved with this project can have access to this information. We can disclose the information only with your permission, except as required by law. In accordance with relevant Australian privacy and other relevant laws, you have the right to access and correct the information we collect and store about you. Please contact us if you would like to access this information. The study information will be re-identifiable. This means that we will remove your child’s name and give the information a special code number. Only the research team can match your name to their code number, if it is necessary to do so. When we write or talk about the results of this project (e.g. at a conference, in a thesis, or a paper), we will report information about the whole group of participants only. This means that no one will be able to identify you.

All study information will be stored securely in a locked filing cabinet in the School of Psychological Sciences at Monash University. Your information and contact details will also be stored on a restricted access, password-protected computer database. The information may be disposed of in a confidential manner as per law.

1. **How is my data protected when I interact with the voice assistant ?**

All your interactions with the voice assistant are encrypted and then securely stored on Amazon’s cloud. That means that your data is encrypted before it reaches the cloud and as a result, when it is stored it cannot be understood without a security key. This is to prevent unauthorized access to, or disclosure of, your content. The recordings that are captured by Amazon are not accessible by either Monash or CogniVocal. However, Monash and CogniVocal may capture certain phrases and sentences spoken to the voice assistant related to the activities you may complete. Participants can review and listen to all recordings and can manually purge them from the system (see below).

1. **How does the voice assistant work?**

The voice assistant you receive is designed to detect only your chosen wake word (Alexa, Amazon, Computer, or Echo). By default, the wake word is ‘Alexa’. After the wake word is activated, a visual indicator appears on your speaker device. For your device, the light ring on the speaker will turn blue and only then will audio (of what you say to Alexa) be sent to the Amazon cloud to process your request.

1. **Can the voice assistant capture unintended conversations?**

Yes. Although the voice assistant does not record all your conversations, it does start recording after it is activated by hearing the word, ‘Alexa’.

1. **How can I mitigate the risk of the voice assistant capturing unintended conversations?**

You can mute the speaker and disconnect the power to the microphones (red light will be illuminated). When the microphones are turned off, your device cannot stream audio to the Amazon cloud, even when you say your chosen wake word.

You can visit the following website link, <https://www.amazon.com/alexaprivacysettings>, and review and listen to your voice recordings and delete all voice recordings associated with your account.

The following site authored by Amazon is intended to provide further clarity and details about how you can protect your privacy and instructions on how to activate the features and controls such as muting the device or deleting all recordings.

<https://www.amazon.com/Alexa-Privacy-Hub/b?ie=UTF8&node=19149155011>.

1. **Can data transmitted to the Amazon cloud identify who I am?**

The only user specific data transmitted to the Amazon cloud is a unique identifier assigned to your user account and device. This does not contain any of your Personal Identifiable Information (PPI) so even if someone were to obtain this unique identifier, they will not be able to identify you. However, because recordings are transmitted, again they are protected by data encryption, there is a risk that what you say contains personal identifiable information. E.g. you say your name, address etc.

1. **Will we be informed of the results when the research project is finished?**

You will receive an email newsletter at the completion of the project that will update you on how the study results and future research direction. A summary of your results will be provided to you if requested

The research findings may be published in journal articles and conference proceedings. All data used for this purpose will be de-identified and analysed as a group to protect the privacy of participants.

1. **Will I be reimbursed for participating?**
2. Phase 1: You will receive a $20 Coles-Myer gift card for your time in testing the voice application and answering the survey regardless of whether you participate in the optional focus group.**Who should I contact for more information?**

If you would like more information about the project, please contact:

| Name: | Sally Richmond |
| --- | --- |
| Contact telephone: | 9905 3935 |
| Email: | [sally.richmond@monash.edu](mailto:sally.richmond@monash.edu) |

| Should you have any concerns or complaints about the conduct of the project, you are welcome to contact the Executive Officer, Monash University Human Research Ethics Committee (MUHREC):  Executive Officer Monash University Human Research Ethics Committee (MUHREC)  Room 111, Chancellery Building D, 26 Sports Walk, Clayton Campus Research Office Monash University VIC 3800  Tel: +61 3 9905 2052 Email: [muhrec@monash.edu](mailto:muhrec@monash.edu) Fax: +61 3 9905 3831 |
| --- |

**Figure S2: Phase 1 installation instructions**

**Set Up Your Access to the Voice Assistant**

You can access the voice assistant, called *Emotion Coaching Study*, using your phone or a home speaker.

**1. Download the Alexa app and sign in.**


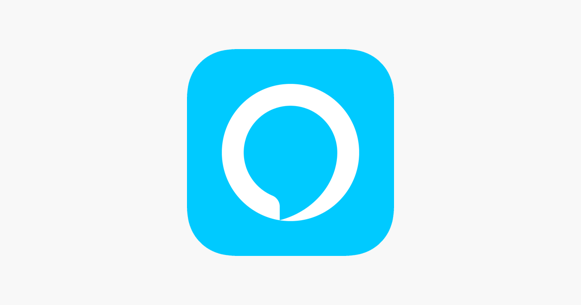


With the free Alexa app, you can set up your device, manage your alarms, music, shopping lists, and more.

The Alexa app is available on phones and tablets with:

• Fire OS 2.0 or higher

• Android 4.0 or higher

• iOS 7.0 or higher

To download the Alexa app, go to the app store on your mobile device and search for "Alexa app." Then select and download the app.

You can also select a link below:

• Apple App Store

• Google Play

• Amazon Appstore

You can also go to https://alexa.amazon.com from Safari, Chrome, Firefox, Microsoft Edge, or Internet Explorer (10 or higher) on your Wi-Fi enabled computer.

To get started using Alexa within the app, tap the Alexa icon at the bottom. Tap **Allow** to bring up the prompt to grant the Alexa app access to your device's microphone and location data. The app will then give you some example commands. Tap **Done** to complete setup.

**2. Register to Beta Testing**.

Once you have registered via the Alexa app we will register you as a beta tester for the **Emotion Coaching Study** skill.

If the email address you use to register for the Alexa app is different to the one you have used to contact the research team please notify the study co-ordinator, Sally Richmond (sally.richmond@monash.edu).

You will receive an email from CogniVocal inviting you to test a new Alexa skill.

Please click on the link underneath the text: To get started, follow this link: [Enable Alexa skill "Emotion Coaching Study"](https://www.amazon.com/gp/f.html?C=1V2NT2IPUYAGD&M=urn:rtn:msg:20200718042646df1037cf1f084a5f89aa804eb560p0na&R=37M90TMLDDK2V&T=C&U=https%3A%2F%2Fskills-store.amazon.com%2Fdeeplink%2Ftvt%2Fab8218917976c25a54678cc8a231f5d3c5f20683637c35c827d8db630145db059169024b0fc2e49b5323ac91d4a36b82b26752d40c77e2058ee581215ae4a798d3dd735c401a0ebeca38060c5698c0249ccdd09d47f26fc0ae16b283f5a489581370ceac277800e3254ecf95f71764%3Fref_%3Dpe_2969780_227219400&H=TQPSJRVLSF4TSPAH0PAYBHUAQDSA&ref_=pe_2969780_227219400)

Tapping the Alexa button will now bring up the voice assistant and you can issue voice commands just as you would with the Alexa speakers around your home. To open the Emotion Coaching Study App on your phone, say “Open Emotion Coaching Study”.

Follow the instructions below to use your home speaker. The example below is for an Echo Dot.

**3. Turn on Echo Dot.**

Place your Echo Dot in a central location (at least eight inches from any walls and windows).

Then, plug the included power adapter into Echo Dot and then into a power outlet.

The light ring on Echo Dot turns blue, and then orange.

When the light turns orange, Alexa greets you.

Note: Other USB power adapters, like phone chargers, may not provide enough power to Echo Dot.

**4. Connect Echo Dot to a Wi-Fi network.**

In the Alexa app, follow the instructions to connect Echo Dot to a Wi-Fi network.

To learn more, go to:

• Connect Echo Dot to Wi-Fi

Tips:

• If the setup process does not automatically start, press and hold the Action button on Echo Dot for five seconds. Then, open the Alexa app, and go to Settings > Set up a new device.

• If Echo Dot doesn't connect to your Wi-Fi network, restart Echo Dot by unplugging and plugging the device in again. If you still have trouble, reset your Echo Dot to its factory settings and set it up again.

**5. Talk to Alexa**.

You can now use your Echo device.

To get started, say the "wake word" and then speak naturally to Alexa.

Your Echo device is set to respond to the wake word "Alexa" by default, but you can use the Alexa app to change the wake word at any time.

In the app, go to Settings, select your Echo device, and then select Wake word.

To open the Emotion Coaching Study App on your speaker, say “Alexa, Open Emotion Coaching Study”.

**Fun Activities to Try.**

Ask Alexa to play music, answer questions, find recipes, read the news, check the weather, set alarms.

Remember that if you are using your phone you need to tap on the blue Alexa button before you speak.


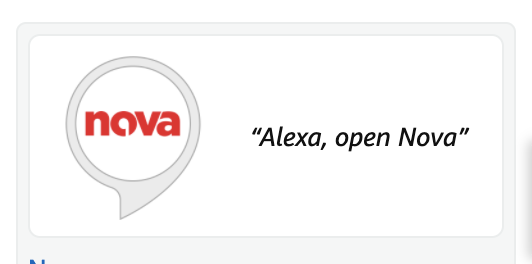


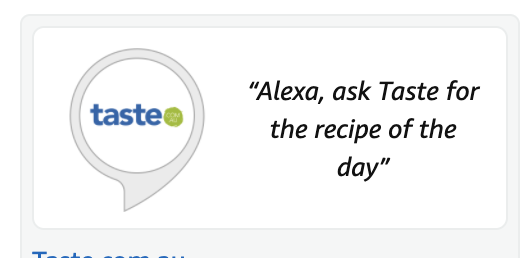


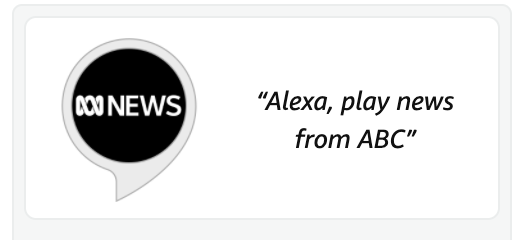

Supplement: Supplementary file 1 [file Table_1.DOCX]
